# Relationship between Serum Soluble Klotho Protein and Coronary Artery Calcification and Prognosis in Patients on Maintenance Hemodialysis

**Published:** 2018-04

**Authors:** Shubei ZHENG, Yu ZHENG, Lingwei JIN, Zhihong ZHOU, Zhanyuan LI

**Affiliations:** Dept. of Nephrology, The Second Affiliated Hospital & Yuying Children’s Hospital of Wenzhou Medical University, Wenzhou, Zhejiang, China

**Keywords:** Kidney dialysis, Soluble Klotho protein, Coronary artery calcification, Mortality

## Abstract

**Background:**

We aimed to investigate the relationship between serum soluble Klotho protein (sKlotho) level and coronary artery calcification (CAC) as well as prognosis in patients with maintenance hemodialysis (MHD).

**Methods:**

Overall, 128 adult patients with end-stage renal failure treated with MHD were collected in the Second Affiliated Hospital & Yuying Children’s Hospital of Wenzhou Medical University, Zhejiang Province, China in 2013. Serum sKlotho was detected by ELISA and coronary artery calcification was measured by multi-slice spiral computed tomography (MSCT). With 36 months’ follow-up, death notes such as cause of death and death time were recorded.

**Results:**

Patients were divided into low sKlotho group and high sKlotho group. Age, blood phosphorus level, hypertension incidence and incidence of diabetes mellitus of the patients in low sKlotho group was significantly higher than that of high sKlotho group (*P*<0.05). The coronary artery calcification score (CACs) of patients in high sKlotho group was significantly lower than that of low sKlotho group (*P*<0.001). Logistic regression showed that the decrease of sKlotho level (*P*<0.001) was an independent risk factor for CAC progression. The mortality of the patients in low sKlotho group was higher than that of high sKlotho group. Kaplan-Meier survival curve had shown that survival time of the patients in low sKlotho group was significantly lower than that of high sKlotho group (*P*<0.05).

**Conclusion:**

SKlotho can increase the degree of CAC. Although MHD patients with low sKlotho level had shorter survival time, sKlotho is not an independent risk factor in prediction of prognosis of MHD patients.

## Introduction

The discovery of the anti-aging gene, Klotho in 1997 gave rise to a new area of research (1). The human Klotho gene, which contains five exons, is localized on chromosome 13q12. Two protein products are produced from the Klotho gene by selective splicing, a membrane-bound form consisting of 1012 amino acids, and a secreted form consisting of 549 amino acids (2). The membrane form of Klotho is a transmembrane protein whose extracellular portion can be cleaved and removed by secretase. The resulting protein fragments and secreted Klotho proteins are collectively referred to as soluble Klotho proteins (sKlotho) (3). sKlotho, which is the primary form of Klotho in blood (4), has a variety of biological activities, such as regulation of energy metabolism. In addition, it has anti-inflammatory and antioxidant properties, and can modulate ion transport and regulate mineral metabolism (5).

The Klotho gene is only expressed in organs such as the kidneys, choroid, and reproductive organs. It is strongly expressed in renal tubular epithelial cells (6). Because of chronic renal failure, gene expression and function of the kidney in patients undergoing maintenance hemodialysis (MHD) are severely affected and renal Klotho mRNA level is significantly reduced. In addition, sKlotho level is significantly reduced (7). Decreased sKlotho level can aggravate vascular calcification in patients on MHD (8), resulting in a significant increase of all-cause mortality and mortality from cardiovascular diseases (9).

The aim of this study was to investigate the relationship between sKlotho level and coronary artery calcification (CAC), all-cause death, and death from cardiovascular disease in patients with MHD, and to investigate further the effect of sKlotho on the prognosis of MHD patients.

## Methods

### Patients

Overall, 128 patients with end stage renal failure who underwent MHD treatment for over 12 months from December 2013 to December 2016 were enrolled in this study. Among them, 52 were female and 76 were male. The ages ranged from 26–92 yr old, with average age of 61.91 ± 15.39 years. The time of dialysis ranged from 25–229 months, with median time of 48 months.

The primary disease was chronic glomerulonephritis in 58 cases, diabetic nephropathy in 34 cases, hypertension renal damage in 25 cases, polycystic kidney disease in seven cases, gouty kidney disease in two cases, and unknown cause in two cases. Exclusion criteria: 1. The primary disease was autoimmune disease; 2. Patients with a history of renal transplantation; 3. Patients with malignant tumors; 4. Patients with acute infection; 5. Patients with severe liver and lung disease; 6. Patients with severe malnutrition; 7. Patients with mental illness or other factors that prevented examination; 8. Patients who underwent coronary artery bypass grafting or coronary stenting; 9. Patients who underwent parathyroidectomy; 10. Patients who died within 3 months after enrollment.

All patients were treated with bicarbonate hemo-dialysis using a Gambro dialysis machine. The dialyzer was Polyflux 14L. All patients were treated three times per week for 4 h each time. Vascular access was an autologous arteriovenous fistula or a jugular vein long-term tube. Blood flow was 250–300 ml/min, dialysate flow was 500 ml/min, and dialysate calcium concentration was 1.50 mmol/l. Each dialysis ultrafiltration volume achieved a clinically assessed dry weight.

This study was approved by the ethics committee of the Second Affiliated Hospital & Yuying Children’s Hospital of Wenzhou Medical University and all patients signed the informed consent.

### General parameters

General parameters including birth date, sex, primary disease, dialysis time since enrollment, body weight, height, and body mass index (BMI) were recorded. In addition, information on combined diseases such as diabetes and hypertension were recorded. Within the first 3 months from the beginning of this study, supine blood pressure of patients was recorded before each dialysis, pulse pressure was calculated, and the average values were recorded.

### Laboratory indexes

During the first 3 months after the beginning of this study, fasting venous blood was extracted from all patients before dialysis. The levels of hemoglobin, albumin, blood glucose, triglycerides, total cholesterol, high-density lipoprotein cholesterol, low-density lipoprotein cholesterol, blood calcium, blood phosphorus, parathyroid hormone, and C-reactive protein were measured. Calcium and urea removal indexes (Kt/V) were calculated and corrected, and the average value of each index was recorded. Serum concentration of sKlotho was determined using an ELISA kit (R&D Co., USA) according to the manufacturer’s instructions.

### Determination of the degree of CAC

CAC scores (CACs) were assessed by multi-slice spiral computed tomography to determine the degree of CAC (10). The scan was performed using a Philips Brilliance 16-slice spiral CT. Before scanning, patients held their breath. The heart rate of patients was controlled at less than 90 beats/min. Patients were in the supine position, first the head was moved in, and the ECG gating devices were connected. Flip-over type ECG gating was used. An aortic root to apical scan was completed within a single breath-hold. Gating settings: Trigger scan point was set at 75% of the ECG RR interval. The images were mainly obtained during cardiac diastole. The scanning mode was 3 mm × 1.5 mm, and scanning length was 144 mm. Ninety six layers were scanned. The scanning conditions were 120 Kv and 55 mA/s, with a matrix of 512 × 512, field of view of 260 mm, and pixel of 0.26 mm. All images were denoised and deartifacted. The reconstructed data were sent to the Vitrea II.fx dedicated graphics processing workstation, and the corresponding software system was used for postprocessing. The degree of CAC was quantified by the Agatston score (11). They were classified as calcified lesions if the area of calcification was not smaller than 1 mm^2^, and the CT peak was not lower than 130 HU.

Calcification of CT values between 130 and 199 Hu was defined as 1; calcification between 200 and 299 Hu was defined as 2; calcification between 300 and 399 Hu was defined as 3; and calcification of CT values above 400 Hu was defined as 4. CACs for each calcified lesion were calculated as follows: CT peak value × corresponding calcification area (mm^2^). Each tomographic image was analyzed independently. The total score obtained by adding the calcification scores of all the faults was the CACs of the patient. The vessels of interest included the left main coronary artery, the left anterior descending artery, the left circumflex artery, and the right coronary artery. The test was completed within 3 months after enrollment.

### Follow-up

The end of follow-up was either December 2016 or the death of the patient. All 128 patients were treated with hemodialysis until the end of the study, and none was switched to peritoneal dialysis. Complications such as hypertension, hyper-glycemia, hyperlipidemia, calcium and phosphorus metabolism disorders, and anemia were controlled by appropriate measures (including drug and non-drug treatment) in accordance with the patient’s condition and relevant treatment guidelines. Cause of death and time of death were recorded according to medical history.

### Statistical analysis

SPSS 19.0 software (Chicago, IL, USA) was used for data analysis and processing. Numerical data of normal distribution are expressed as mean ± standard deviation, and t-test was used for comparisons between two groups. Numerical data of non-normal distribution are expressed as median value (1/4, 3/4). Mann-Whitney U test was used for comparisons between two groups. Categorical data are presented as number of cases and percentage (%), and a χ^2^-test was used for comparisons between two groups. Spearman correlation analysis was used to investigate the correlation between sKlotho levels, baseline indicators, and the degree of CAC. Logistic regression analysis was performed to analyze the risk factors of CAC. Kaplan-Meier survival curves were constructed, and the Cox proportional hazards model was used to analyze the risk factors of prognosis (all-cause death and death from cardiovascular diseases). *P*<0.05 was considered statistically significant.

## Results

### Correlation between sKlotho levels and baseline indicators

The mean value of serum sKlotho level of MHD patients was 401.96 ± 58.59 ng/l, and the patients were divided into two groups according to the mean value: the low sKlotho group (with mean sKlotho level of 357.74 ± 29.08 ng/l) and high sKlotho group (with mean sKlotho level of 455.34 ± 36.37 ng/l). Compared with the high sKlotho group, the patients with low sKlotho had significantly higher age, blood phosphorus, incidence of hypertension, and incidence of diabetes (*P*<0.05) (Table 1).

**Table 1:** Comparison of baseline parameters between the low sKlotho group and high sKlotho group

***Variable***	***Total (n=128)***	***Low sKlotho group (n=70)***	***High sKlotho group (n=58)***	**P *value***
Age (yr, x±s)	61.91±15.39	65.51±13.72	57.55±16.25	0.003
Female [case (%)]	76(59.38%)	45(64.29%)	31(53.45%)	0.214
Smoker [case (%)]	45(35.16%)	28(40.00%)	17(29.31%)	0.207
Combined diabetes [case (%)]	48(37.50%)	33(47.14%)	15(25.86%)	0.013
Combined hypertension [case (%)]	113(88.28%)	68(97.14%)	45(77.59%)	0.001
Phosphorus (mmol/l,x±s)	1.76±0.56	1.87±0.56	1.63±0.53	0.017
C-reactive protein [mg/l,M(1/4,3/4)]	3.90(2.13,8.00)	4.10(2.48,8.95)	3.40(2.03,7.48)	0.169
CACs[M(1/4,3/4)]	45.47(0.00,575.13)	487.57(54.65,965.07)	0.00(0.00,16.67)	0.000

Spearman correlation analysis showed that low sKlotho (sKlotho level < 401.96 ng/l) was positively correlated with age, serum phosphorus, incidence of combined hypertension, and incidence of combined diabetes mellitus (r=0.237, 0.189, 0.303, and 0.219, respectively; *P*=0.007, 0.032, 0.001, and 0.013, respectively); The logistic regression (forward: condition) showed that increases in age ((OR=1.054, 95%CI 1.022–1.086, *P*=0.001), blood phosphorus (OR=1.497, 95%CI 1.150–1.949, *P*=0.003), and incidence of combined hypertension were independent risk factors for low sKlotho in MHD patients

### The correlation between sKlotho and CAC

The CACs of the 128 patients ranged from 0–13450.20, with median CACs value of 45.47 (0.00, 575.13). The CACs of patients in the high sKlotho group [0.00(0.00, 16.67)] were significantly lower than those of patients in the low sKlotho group [487.57(54.65, 965.07)] (*P*=0.000). According to the Rumberger CAC grading method (12), CACs values from 0–10 were classified in the non-calcification group; CACs values from 11–100 were classified in the mild calcification group; CACs values from 101–400 were classified in the moderate calcification group; CACs values above 400 were classified in the severe calcification group. Spearman correlation analysis showed that the degree of CAC was negatively correlated with sKlotho level (r= −0.667, *P* =0.001); Ordered multinomial logistic regression showed that the increase in age (OR=1.077, 95%CI 1.024–1.133, *P* =0.004), increase in time on dialysis (OR=1.027, 95%CI 1.008–1.047, *P* =0.006), and decrease in sKlotho level (OR=1.033,95%CI 1.020–1.044, *P* =0.000) were independent risk factors for CAC.

### The correlation between sKlotho level and prognosis

During the follow-up period, 12 patients were transferred to other hospitals, two patients underwent renal transplantation, and 33 patients died. The annual mortality rate was 9.46%. Among the 33 deaths, 26 died from cardiovascular diseases, two died from tumors, and five died from infection. The all-cause mortality and mortality from cardiovascular disease of patients in the low sKlotho group were higher than those of the high sKlotho group (*P* <0.05) (Table 2).

**Table 2:** Information on sequelae from MHD patients: n(%)

***Variable***	***Total (n=128)***	***Low sKlotho group (n=70)***	***High sKlotho group (n=58)***
Survival	81(63.28)	40(57.14)	41(70.69)
Death	33(25.78)	23(32.86)	10(17.24)
Death from cardiovascular	26(20.31)	19(27.14)	7(12.07)
Death from tumors	2(1.56)	1(1.43)	1(1.72)
Death from infection	5(3.91)	3(4.29)	2(3.45)
Lost access	14(10.94)	7(10.00)	7(12.07)
Kidney transplant	2(1.56)	0(0.00)	2(3.45)
Transferred to other hospitals	12(9.38)	7(10.00)	5(8.62)

**Fig. 1:**
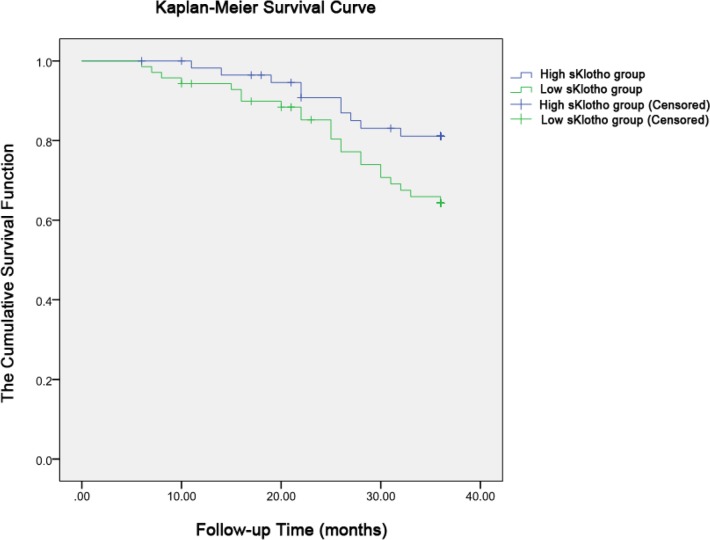
Kaplan-Meier plots of all-cause mortality (*p*=0.047 by log-rank test). The patients were categorized into the low sKlotho (357.74 ± 29.08 ng/l) and high sKlotho (455.34 ± 36.37 ng/l) groups

The Kaplan-Meier survival curve (log-rank test) of all-cause death was constructed for both the low sKlotho group and high sKlotho group. The mean survival time of the high sKlotho group was 33.52 months, while that of the low sKlotho group was 31.31 months. There was a significant difference between the two groups (*P* =0.047) (Fig. 1).

Univariate Cox regression analysis showed that age, incidence of combined diabetes mellitus, and sKlotho levels had significant effects on all-cause mortality in MHD patients (Table 3). Multivariate Cox regression analysis showed that age (HR=1.050, 95%CI 1.019–1.082, *P*=0.002) and combined diabetes mellitus (HR=2.811, 95%CI 1.390–5.684, *P*=0.004) were independent risk factors for all-cause mortality in MHD patients.

**Table 3:** Univariate Cox regression analysis of all-cause mortality in MHD patients

***Parameter***	***B***	***Wald***	**P** ***value***	***HR***	***95.0%CI***	
					***Lower part***	***Upper part***
Age(yr)	.046	10.778	.001	1.047	1.019	1.076
Combined diabetes mellitus	1.090	9.309	.002	2.975	1.477	5.991
sKlotho	−.008	5.976	.015	.992	.985	.998

Kaplan-Meier plots (log-rank test) were constructed for death caused by cardiovascular diseases. The mean survival time was 34.24 months in the high sKlotho group, and 31.90 months in the low sKlotho group (*P*=0.036).

**Fig. 2:**
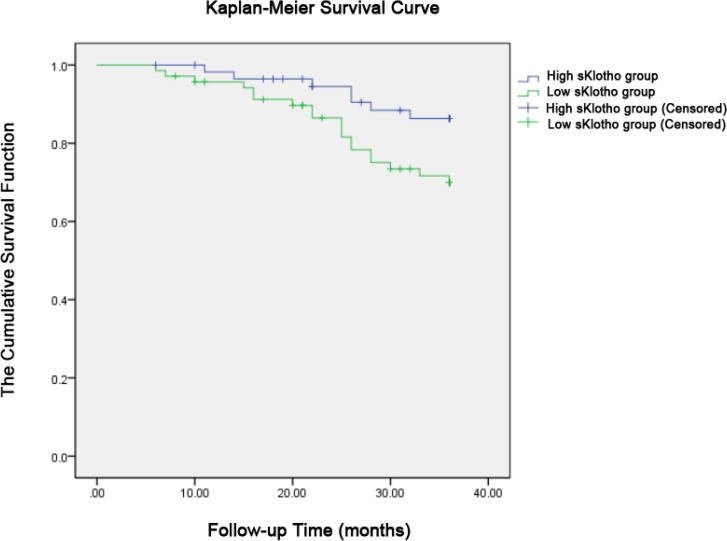
Kaplan-Meier plots of cardiovascular mortality (*p*=0.036 by log-rank test). The patients were categorized into the low sKlotho (357.74 ± 29.08 ng/l) and high sKlotho (455.34 ± 36.37 ng/l) groups

There were significant differences between the two groups (Fig. 2).

Univariate Cox regression analysis showed that age and the incidence of combined diabetes mellitus had significant effects on mortality from cardiovascular disease in MHD patients (Table 4). Multivariate Cox regression analysis showed that age (HR=1.056, 95%CI 1.019–1.094, *P*=0.003) and combined diabetes mellitus (HR=3.398, 95%CI 1.504–7.678, *P*=0.003) were closely related to mortality from cardiovascular disease.

**Table 4:** Univariate Cox regression analysis of mortality from cardiovascular disease in MHD patients

***Items***	***B***	***Wald***	**P** ***value***	***HR***	***95.0%CI***	
					***Lower part***	***Upper part***
Age(yr)	.050	9.660	.002	1.051	1.019	1.085
Combined diabetes mellitus	1.274	9.512	.002	3.576	1.591	8.036

## Discussion

In this study, serum sKlotho level and the age of MHD patients were closely related. With the increase of age, sKlotho levels decreased significantly, and this result was consistent with the previous observation (13). We also found that serum sKlotho levels of patients on MHD were negatively related to serum phosphorus levels. sKlotho could activate extracellular signal-regulated kinase, and stimulate osteoblasts to generate FGF23 (14). The expression of NPt-2a and NPt-2c in renal proximal tubule epithelial cells was reduced by FGF23, which further reduced the reabsorption of phosphorus from urine to increase the excretion of urinary phosphorus. FGF23 can also downregulate 1-hydroxylase to decrease the synthesis of 1,25(OH)2VitD3, and upregulate 24-hydroxylase to promote the degradation of 1,25(OH)2VitD3 to vitamin D3-23 carboxylic acid, and further reduce the intestinal absorption of phosphorus (15). sKlotho can directly inhibit the renal sodium and phosphorus co-transporter, NPt-2a, and reduce urinary phosphorus absorption to decrease phosphorus level, suggesting that sKlotho also has a direct function in phosphorus reduction that is independent from the function of FGF23 (14).

sKlotho plays a pivotal role in the pathogenesis of hypertension (16). We also found that patients with low sKlotho had a significantly higher incidence of hypertension. sKlotho can reduce peroxide-induced endothelial cell apoptosis, increase vascular endothelial cell activity, and decrease the activity of caspase-3 and caspase-9 (17), thereby inhibiting the development of hypertension. Blood glucose and insulin levels decreased in Klotho gene knockout mice, although insulin sensitivity increased (18), suggesting that Klotho is closely related to the incidence of diabetes. Nie et al. (19) found that serum sKlotho levels of patients with type II diabetes were significantly lower than in the normal control group. Our data suggested that the increased prevalence of diabetes in patients with low sKlotho level might be related to the regulatory roles of sKlotho in insulin/insulin-like growth factor-1 (IGF-1) signaling pathways.

CAC is a common cardiovascular complication in patients on MHD, and is the pathological basis of the high incidence of cardiovascular disease. In our patients, sKlotho levels were significantly reduced with increased severity of CAC. Logistic regression suggested that sKlotho reduction was an independent risk factor for CAC. CAC is a series of complex molecular and cellular changes in the intima of the coronary arteries resulting from a variety of etiologies, originating from endothelial cell injury. sKlotho can promote endothelial nitric oxide synthesis, protect endothelial cells, and reduce the degree of atherosclerosis (6). Serum sKlotho levels were highly related to atherosclerosis, oxidative stress, and endothelial cell dysfunction. Increased sKlotho may reduce cardiovascular disease (20).

Based on data over the 36 months follow-up period in 128 MHD patients, we found that all-cause mortality in the high sKlotho group was lower than in the low sKlotho group. The Kaplan-Meier survival curve suggested that patients with high sKlotho had a significantly longer survival time.

Otani-Takei et al. (9) measured serum sKlotho levels in 63 MHD patients, and divided the patients into low, medium, and high sKlotho groups based on sKlotho level. Follow-up of 65 months showed that sKlotho levels in the 12 patients who died were significantly lower than in the surviving patients. Kaplan-Meier survival curves showed that patients with low sKlotho level also had low cumulative survival rate. They also found that age and low sKlotho levels were independent risk factors for all-cause mortality.

In our study, although univariate Cox regression analysis showed that sKlotho reduction was a risk factor for all-cause mortality, while multivariate Cox regression analysis did not suggest that sKlotho was an independent risk factor for poor prognosis. After analysis of the experiments performed by Otani-Takei et al. (9), we found that the patients in their study were selected with a good balance of clinical and demographic baseline parameters. However, the differences in age, blood phosphorus levels, incidence of hypertension, and incidence of diabetes mellitus between the different sKlotho groups in our study may have affected the results. In future studies, the number of enrolled patients should be increased, and the influencing factors should be balanced to investigate further the relationship between sKlotho level and prognosis of MHD patients.

Aging is recognized as an independent risk factor for prognosis, and this was further confirmed by our results. As a coronary artery disease risk equivalent, diabetes can lead to increased levels of sugar oxidation products, glycosylated products, and lipid oxidation production. These in turn lead to further vascular endothelial dysfunction, increased oxidative stress, and accelerated progression of atherosclerosis, eventually affecting prognosis. Our study also found that diabetes mellitus is an independent risk factor for poor prognosis in MHD patients.

## Conclusion

sKlotho levels in MHD patients are associated with age, serum phosphorus, hypertension, and diabetes mellitus. Patients with low sKlotho levels also had severe CAC. In addition, the all-cause mortality of patients in the low sKlotho group was significantly higher than that of patients in the high sKlotho group. However, the results of this study did not suggest that sKlotho is an independent risk factor for poor prognosis.

## Ethical considerations

Ethical issues (Including plagiarism, informed consent, misconduct, data fabrication and/or falsification, double publication and/or submission, redundancy, etc.) have been completely observed by the authors.
